# Reintroducing apex predators: the perils of muddling guilds and taxocenoses

**DOI:** 10.1098/rsos.180567

**Published:** 2018-07-11

**Authors:** Everton B. P. Miranda

**Affiliations:** 1 ONF Brasil Gestão Florestal, Cotriguaçu, MT, Brazil; 2 Universidade do Estado de Mato Grosso, Alta Floresta, MT, Brazil

Wolf & Ripple [1] make the case for ‘large carnivore' conservation through reintroductions. The selected species, however, were neither necessarily apex predators nor carnivores, but a subset of the mammalian order Carnivora, including those ranging from insectivores–omnivores (sloth bear, *Melursus ursinus* [2,3]; sun bear, *Helarctos malayanus* [4]) to herbivores–omnivores (Andean bear, *Tremarctos ornatus* [3,5]; American black bear, *Ursus americanus* [6]), full omnivores (Asiatic black bear, *Ursus thibetanus* [7,8]; brown bear, *Ursus arctos* [9,10]) and several hypercarnivores (jaguar, *Panthera onca* [11]; dhole, *Cuon alpinus* [12]; cheetah, *Acinonyx jubatus* [13]). At first, Wolf & Ripple lump together mammalian carnivores (those in the order Carnivora, which may or may not be regular predators), apex predators (which occupy the top of food chains [14]) and any carnivore (which primarily consumes other vertebrates). Further, they mixed guilds (a group of species that exploit similar resources) with taxocenosis (a group of sympatric species sharing a common phylogenetic clade). Consequently, the authors provide a biased assessment of reintroduction priorities based on conservation imperatives that are not restricted to any of these groups, but primarily associated with apex predators. This commentary explores the development of this muddle, which I interpret to be mainly due to a combination of inaccurate use of nomenclature and concepts. Furthermore, the authors make some mistakes for a number of proposed reintroduction sites that proper investigation would have revealed to be inappropriate.

Mammalian carnivores are not alone as apex predators, as the latter comprise a diverse set of terrestrial and aquatic vertebrates on Earth [14], which include (but are not limited to) large raptors and large, predatory reptiles. By equating apex predators with Carnivora mammals, the authors ignore the diverse predator species that have the same potential to: (i) perform predator-related ecosystem functions [15,16]; (ii) create wildlife-viewing tourism opportunities [15,17]; and (iii) reduce species extinction risk by re-establishing extirpated populations and reversing range shrinkage. Furthermore, this diverse array of apex predators is threatened by exactly the same extinction drivers as ‘large carnivores’, namely livestock predation [16,18], perceived risk to humans [19,20], overhunting [21,22] and habitat loss. In temperate and boreal regions, extant, large raptors may now perform reduced functions as top predators [23], while the role of large reptiles may be non-existent [24]. These latitudes contain most of the developed countries, and therefore most of the ongoing scientific research, which helps explain the authors' biased world view of which species qualify as apex predators in ecosystems outside the Northern Hemisphere. Apex predators outside the Northern Hemisphere are, however, characterized by high diversity.

Reptiles such as crocodilians and giant snakes are important predators of the land–water interface on tropical and subtropical realms. Crocodilians—the largest living continental predators on Earth—can control access of terrestrial vertebrates to vital resources such as water [25] and can inflict havoc and significant mortality on terrestrial mammals, with concomitant effects that cascade down into entire aquatic food webs [26]. Pythons and anacondas, on the other hand, prey on terrestrial vertebrates, thus exerting top-down control over their populations [27,28], as do other apex predators [29], and some mammalian Carnivora [30]. Given their lower metabolic rates, reptiles feed much less frequently than do hypercarnivorous mammals [31], but this is compensated for by the extremely high biomass of heterothermic carnivores [22,32]. Physical power is clearly not a problem, because pythons and anacondas are capable of killing and eating ‘large carnivores' such as bears and large cats [33,34]. Like many mammalian carnivores, large, predatory reptiles also kill domestic livestock and are vulnerable to the same sort of retaliatory persecution that affects ‘large carnivores’ [35,36]. Giant snakes regularly kill domestic dogs in urban, peri-urban and rural landscapes in the tropics [19,37,38]—as do the dog-eating leopards *Panthera pardus* of India [39]. The well-known preference that pythons show for eating canids must be taken into account when planning a possible reintroduction of red wolves (*Canis rufus*) into the Everglades National Park; else we risk losing them to *Python molurus* predation. Predatory reptiles are indeed conspicuous elements of apex predator guilds throughout many regions, but frequently are overlooked as the top predator that they are.

Raptors are the Earth's largest aerial predators in tropical, subtropical and temperate landscapes. As do large, predatory reptiles, giant raptors occasionally even snatch offspring of ‘large carnivores' [40,41]. Extant, large-bodied raptors such as harpy eagles (*Harpia harpyja)*, crowned eagles (*Stephanoaetus coronatus)* and Philippine eagles (*Pithecophaga jefferyi)* perform the role of apex predators in the tropical forest canopy [42–44], a habitat that is largely or completely inaccessible to large, terrestrial carnivores. Giant raptors are therefore apex predators in the world's most biodiverse terrestrial ecosystems [45,46]. In tropical forests and other biomes, giant raptors exert the same top-down roles [47–49] of the more widely celebrated ‘large carnivores'. As for wolves, research has documented the cascading effects on diverse trophic levels when apex aerial predators are missing. Clearly, the largest raptors play important roles in maintaining balance in the vertebrate communities of forest canopies [49–51]. Trophic cascade ecology is a research topic that has conspicuously focused on the Carnivora mammal taxocenosis. By contrast, giant raptors have been largely ignored by Wolf & Ripple and by other research reviews on apex predators.

While proposing species reintroductions, Wolf & Ripple provided a superficial examination of the occurrence, habitat suitability and original distributions of ‘large carnivores'. For the sake of brevity, I restrict my comments to their proposed reintroductions in the Neotropics. For example, the first of the six sites suggested for jaguar reintroduction, La Payunia, is a barren scrubland that provides inadequate habitat for jaguars, while the other five sites already harbour jaguar populations, thus obviating the need for reintroduction there. Four of the last five sites, namely Campos Amazônicos [52], Guaporé [53], Pacaás Novos [54] and Mapinguari [55], have published reports already confirming the presence of jaguars, while the final site, Rio Novo, obviously must have jaguars as that protected area is embedded within a vast region of continuous Amazon forest with a low human footprint. Cordillera Azul National Park, where Wolf & Ripple propose the reintroduction of pumas (*Puma concolor*), already has a population of pumas [56]. Similarly, I found weaknesses in the recommended reintroduction sites for Andean bears. Running down that list of six sites, one finds (i) La Tatacoa and Cerro Saroche are arid, rocky landscapes that, while they might support some bears as landscapes (as do Chaparri), are far from good habitat for the species, and hence should not be prioritized; (ii) Serrania de Minas has an Andean bear population discovered recently by local, government-supported researchers [57], and researchers found bears in Pampa Hermosa in Peru [58]; (iii) Tinigua is at approximately 300 m.a.s.l. and offers only marginal habitat for Andean bears, which tend to do best in cloud forests at 2500–3000 m.a.s.l. [59]; and (iv) the occurrence of Andean bears in Panama is at best speculative [60], with the proposed site (Dárien) lacking sufficient elevation [61] and perhaps receiving wandering bears into Panama from Los Katios in Colombia (Bernard Peyton, personal communication).

Therefore, all the reintroductions recommended for the Neotropics are either inappropriate or mistaken. Global-scale studies commonly show weakness when one examines specific cases one by one, but then one wonders how valid conclusions can be drawn from what appear to be such universally incorrect locations in the Neotropics.

When they state that ‘reintroduction efforts would lead to *complete* large carnivore guilds’, Wolf & Ripple ignore many of the Earth's most quintessential predators. Taxonomic chauvinism has long led to a biased body of research that does not lead to a realistic model of the frequency and magnitude of the ecological roles of different species. Their paper is another example of the lack of balance in global analyses of predator ecology. Attention to predators that in fact have more varied feeding habits such as omnivory as well as the need to include non-mammalian predators and the need for reliable global databases on species presence are all essential to the study of ecology. Ignoring these factors results in biased conclusions that are of questionable value in constructing and carrying out conservation policy in this age of extremely scarce funding for conservation.

## Data accessibility

This article has no additional data.
